# Correction: Mitochondrial event localiser (MEL) to quantitatively describe fission, fusion and depolarisation in three-dimensional space

**DOI:** 10.1371/journal.pone.0249162

**Published:** 2021-03-24

**Authors:** The PLOS ONE Staff

There should be a space between the words "quantitatively" and “describe” and the word “the” should be removed in the article title. The correct title is: Mitochondrial event localiser (MEL) to quantitatively describe fission, fusion and depolarisation in three-dimensional space. The correct citation is: Theart RP, Kriel J, du Toit A, Loos B, Niesler TR (2020) Mitochondrial event localiser (MEL) to quantitatively describe fission, fusion and depolarisation in three-dimensional space. PLoS ONE 15(12): e0229634. https://doi.org/10.1371/journal.pone.0229634

The publisher apologizes for the error.
