# Supplementary figures and images for: Mitochondrial event localiser (MEL) to quantitativelydescribe fission, fusion and depolarisation in the three-dimensional space
Source: PLoS One. 2020 Dec 30;15(12):e0229634. doi: 10.1371/journal.pone.0229634 (PMC7773280; doi:10.1371/journal.pone.0229634)

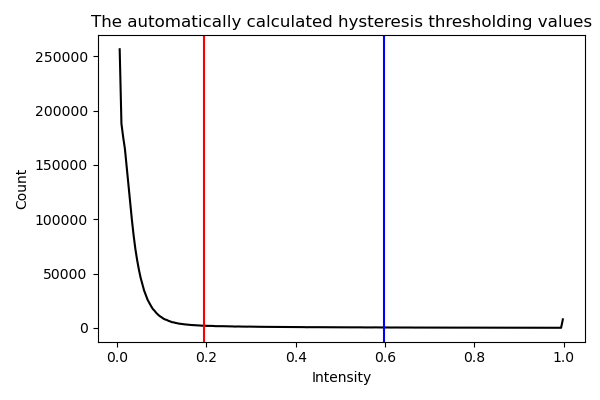

Supplement: S1 Fig — The low threshold is automatically calculated at the edge of the histogram valley of the background voxel intensities, and the high threshold at the halfway point between the low threshold and the maximum intensity. (PNG) [file pone.0229634.s001.png]

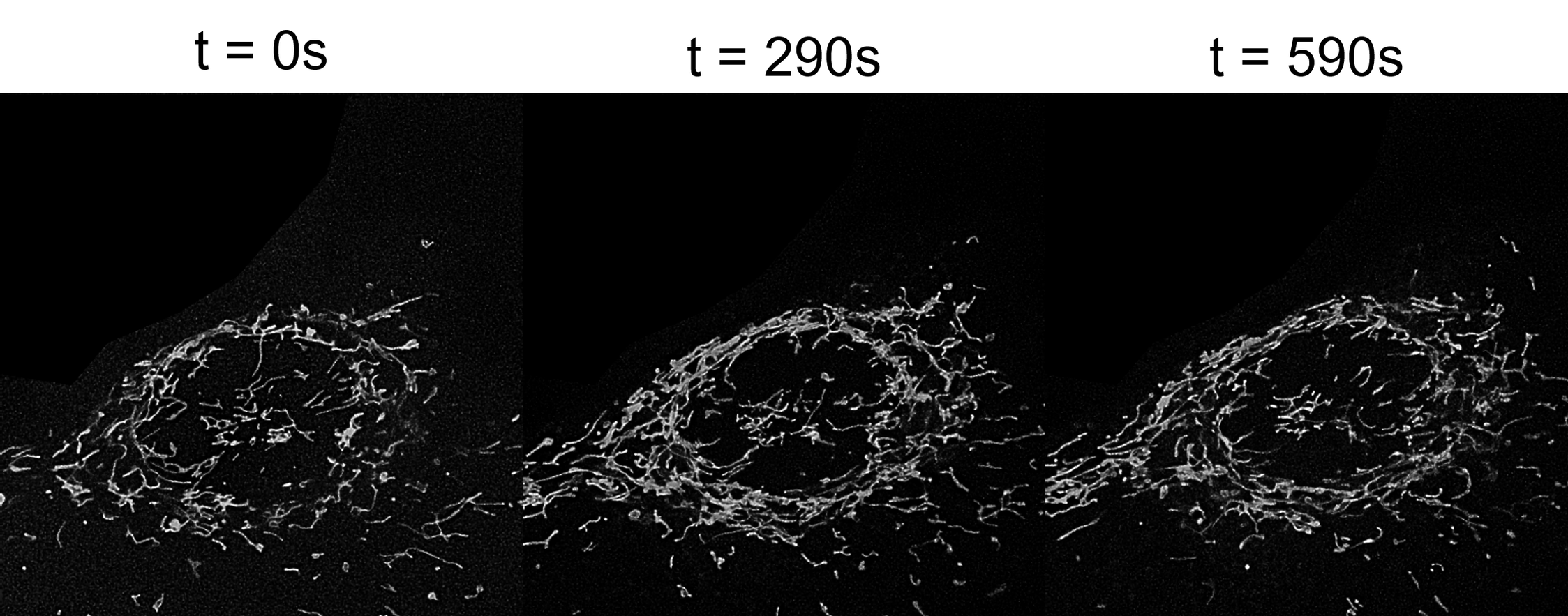

Supplement: S2 Fig — Control cells were imaged every 10 seconds for a total of 10 minutes. In order to illustrate the limited effect of photo bleaching, control cells that were acquired after 0 sec, 290 sec, and 590 sec are compared. (TIF) [file pone.0229634.s002.tif]
