# Supplementary material for: Mitochondrial event localiser (MEL) to quantitativelydescribe fission, fusion and depolarisation in the three-dimensional space
Source: PLoS One. 2020 Dec 30;15(12):e0229634. doi: 10.1371/journal.pone.0229634 (PMC7773280; doi:10.1371/journal.pone.0229634)
Supplement: S1 Appendix — (PDF) [file pone.0229634.s003.pdf]

S1 Appendix: Application of MEL to a synthetic example

Rensu P Theart*, Jurgen Kriel, Andre du Toit, Ben Loos, Thomas R Niesler

* rptheart@sun.ac.za

In this appendix we demonstrate the steps of the MEL algorithm by applying it to two synthetically generated image frames. This is intended only to clarify the MEL algorithm, and does not represent a realistic scenario as would be expected when analysing mitochondrial events. Although MEL is intended for application to three-dimensional samples, we will consider two-dimensional images here for clarity.

Since our images are synthetic, we omit the normalisation step shown in Fig 1 and begin by showing binarised Frames 1 and 2 in Fig A. We also overlay these binarised images to make it easier to identify those structures that will fuse or undergo fission from Frame 1 to Frame 2. Next, each structure in the binarised frames is given a unique label number, with 0 indicating the background. Each labelled structure is then separated to create an array of images (not shown in Fig A) which is Gaussian filtered to allow for a less strict structure overlap matching between Frame 1 and Frame 2. The array of labelled images are also Canny filtered, leaving only the pixels on the edge which is used to determine the distance between structures and the location of the fission and fusion events. Fig A shows only the first labelled structure in the array for each frame. These images are then analysed according to the process in Fig 2 to produce a set of locations and types of mitochondrial events that are hypothesised to have occurred in the time between Frame 1 and Frame 2. The result is then overlaid with Frame 1 using colour labels for the different events.

The matrices and arrays calculated for the synthetic example by the process depicted in Fig 2 are shown in Fig B. The overlap matrix V is calculated by multiplying all combinations of the Gaussian filtered structures, one from Frame 1 and one from Frame 2, to determine a representation of the volume. Label number 0 indicates the background and therefore is left blank throughout.

From matrix V, the arrays A_1 and A_2 are determined by simply reducing V to indicate which structures in the other frame presented with a non-zero overlapping volume. The relative percentage overlap, P_1 and P_2, of each structure in one frame with all associated structures in the other frame can then be calculated from A_1 and A_2. This is in effect a normalisation of the volumes in matrix V, where the volume of a certain structure combination is divided by the total volume of the given structure in either Frame 1 (producing P_1) or Frame 2 (producing P_2). Each row, therefore, sums to 100%.

Using the back-and-forth structure matching described in Fig 3, we generate W_1 and W_2, to indicate which structures are associated with each other in the same frame. These are candidates for fission and fusion events, although some of them could be false matches that the next step aims to eliminate. It is worth noting that each structure combination occurs twice in the array of lists. Even though we show the calculations that follow for both, it is only necessary to use one.

Using W_1 and W_2 along with the edge array of images E_{ar} for Frame 1 and Frame 2, we find the shortest distances, D_1 and D_2, as well as the midway points, M_1 and M_2, between each combination of structures. If the shortest distance between the candidate structures is above a set threshold (in the case of this synthetic sample this was 50 pixels), the two structures are considered unrelated and is ignored in the visualisation (refer to Fig A and Fig C). Secondly, if the midway point is sampled from the binarised

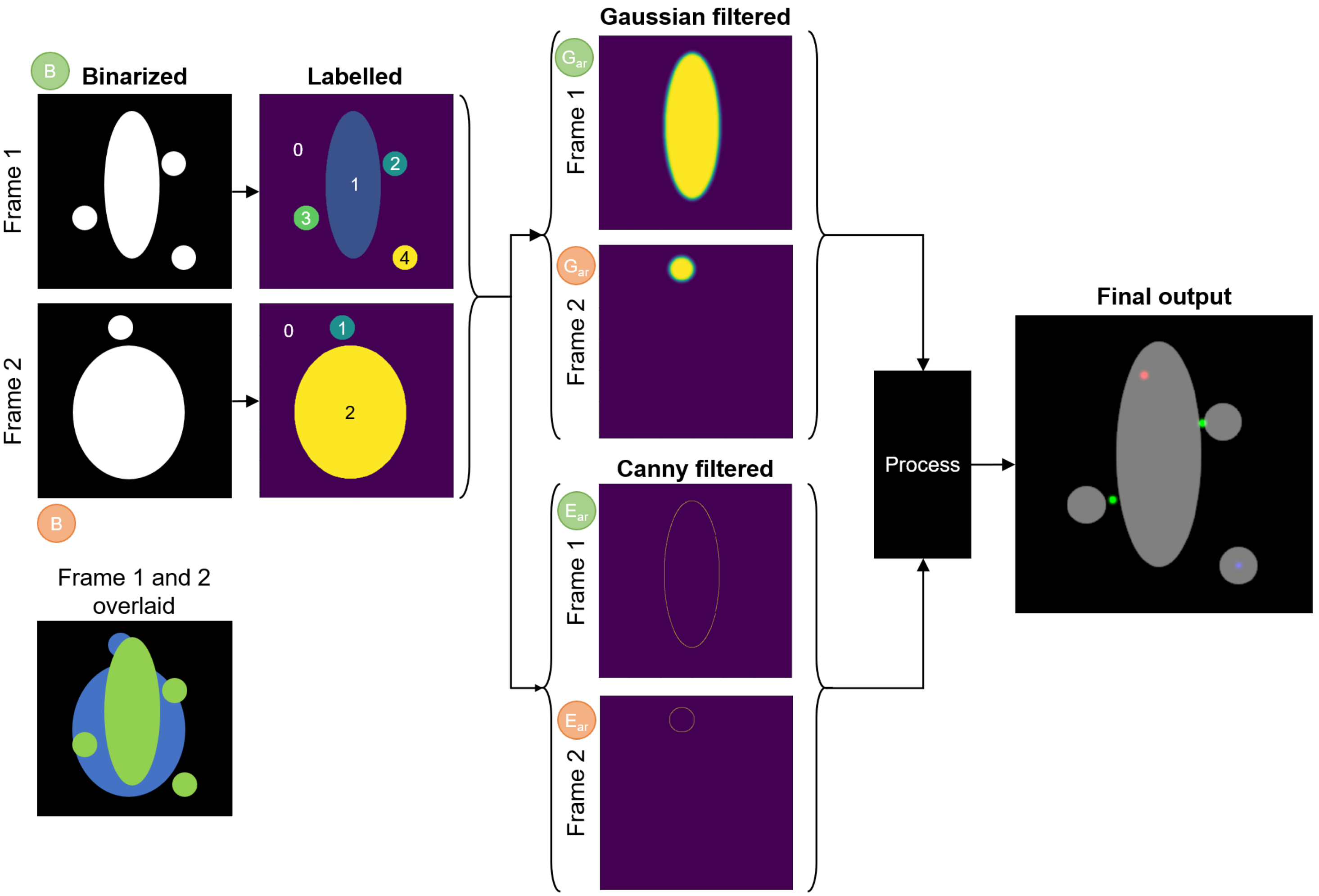

Fig A. Synthetic image example process flow.

images, B_1 and B_2, and coincides with another structure it is also considered a false match. This is because two structures cannot fuse through a third separating structure. Rather, they would individually fuse with the central structure. A similar logic applies when considering fission events in Frame 2. This is illustrated in Fig C for a fusion, fission and unrelated label combination.

Now the mitochondrial event status can be determined for each candidate structure combination in W_1 and W_2. Structures in Frame 1 are labelled "Fuse", "Depolarize", or "Unrelated", while structures in Frame 2 are labelled "Fission", or "Unrelated".

Finally, using the midway points, as well as the centre of mass of the structures to indicate the location of the mitochondrial event, along with the status arrays, the final output image is generated (Fig A).

Matrix of overlapping volumes

V

Frame 1 label no	Frame 2 label no 0	1	2
0			
1		2394	56809
2		0	2396
3		0	4647
4		0	0

Array of lists of associated structures between frames

A_1

Frame 1 label no	Associated label nos in Frame 2	
0		
1	1	2
2	2	
3	2	
4		

A_2

Frame 2 label no	Associated label nos in Frame 1		
0			
1	1		
2	1	2	3

Related arrays of lists

Labels between frames	
A_1	P_1
A_2	P_2

Relative percentage overlap

P_1

Frame 1 label no	Overlap with associated structures in Frame 2	
0		
1	4%	96%
2	100%	
3	100%	
4		

P_2

Frame 2 label no	Overlap with associated structures in Frame 1		
0			
1	100%		
2	89%	4%	7%

Array of lists of associated structures within the same frame

W_1

Frame 1 label no	Associated label nos in Frame 1	
0		
1	2	3
2	1	3
3	1	2
4		

W_2

Frame 2 label no	Associated label nos in Frame 2
0	
1	2
2	1

Array of lists of midway points (MWP) between associated structures

M_1

Frame 1 label no	Associated structures MWP in Frame 1	
0		
1	(223, 389.5)	(1, 382, 203.5)
2	(223, 389.5)	(1, 307.5, 292)
3	(382, 203.5)	(1, 307.5, 292)
4		

M_2

Frame 2 label no	Associated structures MWP in Frame 2
0	
1	(124.5, 269)
2	(124.5, 269)

Array of lists of distances between associated structures

D_1

Frame 1 label no	Distance between two structures in Frame 1	
0		
1	9	29
2	9	251
3	29	251
4		

D_2

Frame 2 label no	Distance between two structures in Frame 2
0	
1	19
2	19

Related arrays of lists

Labels between frames			
W_1	M_1	D_1	Fu-sion
W_2	M_2	D_2	Fis-sion

Assigned status to structure combinations

Fu-sion

Frame 1 label no	Status of label combinations in Frame 1	
0		
1	Fuse	Fuse
2	Fuse	Unrelated
3	Fuse	Unrelated
4	Depolarize	

Fis-sion

Frame 2 label no	Status of label combinations in Frame 2
0	
1	Fission
2	Fission

Fig B. Synthetic image example calculated matrix and list of arrays.

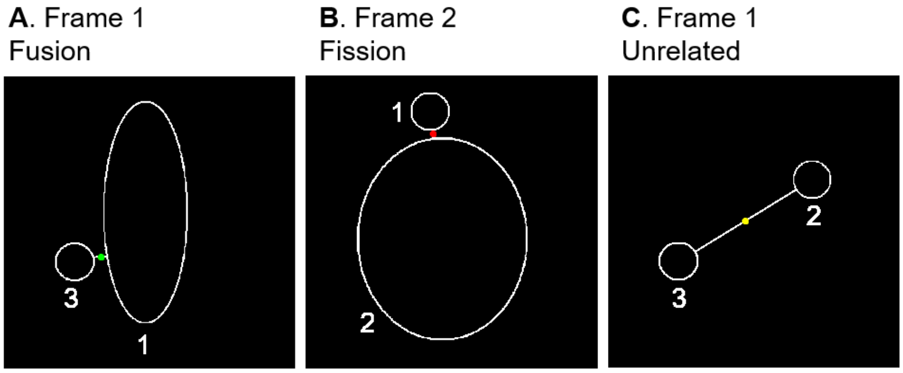

Fig C. Example of determining fission and fusion event locations.
