# Supplementary material for: Mitochondrial event localiser (MEL) to quantitativelydescribe fission, fusion and depolarisation in the three-dimensional space
Source: PLoS One. 2020 Dec 30;15(12):e0229634. doi: 10.1371/journal.pone.0229634 (PMC7773280; doi:10.1371/journal.pone.0229634)
Supplement: S2 Appendix — (PDF) [file pone.0229634.s004.pdf]

S2 Appendix: General considerations when using MEL

Rensu P Theart*, Jurgen Kriel, Andre du Toit, Ben Loos, Thomas R Niesler

* rptheart@sun.ac.za

Due to the motility of mitochondria, it is common to confuse two mitochondria passing each other as a fusion event at one point in the time-lapse and as a fission event at a later point [1]. However, since MEL considers the entire three-dimensional structure of mitochondria, it can distinguish mitochondrial structures passing each other from true fusion between these structures. For this to be possible, the microscope that is used should have sufficient resolving power and the z-stack should contain a sufficient number of micrographs. We found that using a 100x oil immersion objective with the microscope and acquiring image stacks with 4-6 micrographs, with a 0.5 μm step width, produces favourable results.

We recommend the use of a fluorescent probe that achieves favourable signal to noise ratio. Other mitochondrial probes and trackers, or specific mitochondrial proteins, could equally be employed, given that depolarisation events lead to sudden loss in specific mitochondrial signal.

MEL is strongly dependent on the quality of the binarised frames (B_1 and B_2). The ability of the thresholding algorithm to perform well is in turn dependent on the quality of the input images. It is for this reason that we first normalise the images before applying hysteresis thresholding to ensure it produces more consistent results. However, if the input image quality is poor, a small adjustment in pre-processing parameters can have a large impact on the resulting binarised image. The consequence of this is that some structures could be joined together for some pre-processing parameter choices and be binarised as separate structures with others (as illustrated in Fig 5). Furthermore, choosing acquisition parameters to produce with z-stacks with high dynamic range and a good signal-to-noise ratio also improves the accuracy of the binarisation.

MEL has tuneable parameters that affect how it performs. These are the pre-processing parameters as well the percentage and distance thresholds, which are used to remove false matches. Whenever comparative analysis between different cells are performed, it is important to use the same values for these parameters as well as the same acquisition parameters for the microscope. The values of tuneable parameters that were used in our investigation are summarised in Table A. These values were selected based on empirical observation. While we believe that the chosen values for these parameters generalise well, an automated statistical method could also be employed to determine them based on the z-stack under analysis. Such a strategy was used, for example, for an automated image analysis algorithm that determines the optimal filtering parameters to produce an optimised 3D quantification of mitochondrial morphology [2].

Table A. The tuneable parameters used by MEL for our analysis. The noise volume of 40 voxels is equivalent to a circle with a diameter of 5 pixels in the original image, or in our case a circular structure with a physical diameter of about 0.6 μm.

**Tuneable parameter**	**Value**	**μm**
Scale factor	1.5	-
Noise volume	40 voxels	≈ 0.6
Normalisation Gaussian blur (x-y)	$\sigma_{2D} = 1.0$	≈ 0.08
Gaussian blur	$\sigma_{3D} = 0.25$	≈ 0.02 in 2D and ≈ 0.125 in 3D
Relative percentage threshold	50%	-
Distance threshold	100px	≈ 11.86

References

1. Westrate LM, Drocco JA, Martin KR, Hlavacek WS, MacKeigan JP. Mitochondrial morphological features are associated with fission and fusion events. PLoS One. 2014 Apr 14;9(4):e95265.

2. Nikolaisen J, Nilsson LI, Pettersen IK, Willems PH, Lorens JB, Koopman WJ, et al. Automated quantification and integrative analysis of 2D and 3D mitochondrial shape and network properties. PLoS One. 2014 Jul 2;9(7):e101365.
