# Supplementary material for: Mitochondrial event localiser (MEL) to quantitativelydescribe fission, fusion and depolarisation in the three-dimensional space
Source: PLoS One. 2020 Dec 30;15(12):e0229634. doi: 10.1371/journal.pone.0229634 (PMC7773280; doi:10.1371/journal.pone.0229634)
Supplement: Attachment — Submitted filename: Responses to the reviewer (Second revision).pdf [file pone.0229634.s007.pdf]

Responses to the reviewer's comments for the revised submission:

"*Mitochondrial event localiser (MEL) to quantitatively describe fission, fusion and depolarisation in the three-dimensional space*".

PONE-D-20-03864

Theart, Kriel, du Toit, Loos, and Niesler
University of Stellenbosch
South Africa

We would like to thank the reviewers for their effort in reviewing our paper. The comments have been highly constructive, allowing us to consider the details of the method we present more closely and state our design choices more clearly. We have carefully addressed the recommendations.

Response to the editor

In response to the points raised by the editor, in the revised manuscript we have now clarified in line 419 as well as in the caption of Fig 10 that the results are indeed independent, biological repeats. For this reason, we believe that the method is sufficiently validated.

In the following text, reviewers' verbatim comments are shown first, and our responses follow.

Reviewer 2.

Major critiques:

Reviewer's comment 1.

The revision addresses most of my minor critiques but fails to address my primary concern that the authors have not proven the repeatability of the biological findings they are claiming with repeat experimental replicates. While they do describe their n in terms of number of cells analyzed, I cannot find anywhere in the paper that suggests the biological experiment was repeated more than once under identical conditions for each condition. This unfortunately remains a fatal flaw that must be addressed prior to publication. If this is what was done for Fig. 10, please make it abundantly clear to the reader that these were independent experiments.

Authors' response 1.

We wish to thank the reviewer for indicating this important aspect. We have now made it clear in line 419 as well as in the caption of Fig 10, that the results are indeed independent, biological repeats. For each biological repeat, newly passaged cells were seeded and subsequently imaged. Since we utilised 8-chamber dishes, with each independent experiment, also technical repeats were acquired.

Moreover, we are now also referring to additional avenues (line 528) that may be considered in future, to further decrease variability, such as cell cycle synchronisation, or, as established in our laboratory, micropatterning.

Reviewer's comment 2.

Abstract:
The high rate of error should be addressed as a limitation up front so as not to surprise the reader in the results. The authors make the argument that the error of the algorithm in correctly detecting fission fusion and depolarization events in their peroxide-induced stress experiment correlates with the ground truth data as determined by manual review but with false positive rates ranging from at best 84% to 2000% (taken by dividing the means on the left side by the right side on Table 2), this is a huge limitation.

Authors' response 2.

We wish to thank the reviewer for this comment. We have now addressed this limitation in the abstract upfront by adding the following sentence:
"When individually validating mitochondrial events detected with MEL, for a representative cell for the control and treated samples, the true-positive events were 47%/49%/14% respectively for fusion/fission/depolarisation events."

In addition, we have also added a new table (Table 2) to indicate the accuracy for the different conditions, and enhanced clarity in the text (line 470).

Reviewer's comment 3.

Please present the critical quantitative findings of the paper from Fig. 10 in the abstract or consider changing the title from quantitative to qualitative. E.g., "an average of x/x/x fusion/fission/depolarisation events per cell (verified/unverified?) were observed every 10 sec in control cells. With peroxide treatment, the rate initially rebalanced toward fusion to x/x/x, before returning to approximately the same as control levels x/x/x."

Authors' response 3.

We thank the reviewer for this recommendation. We have now presented the critical quantitative findings in the abstract by adding the following text:
"An average of 9.3/7.2/2.3 fusion/fission/depolarisation events per cell were observed respectively for every 10 sec in the control cells. With peroxide treatment, the rate initially shifted toward fusion with an average of 15/6/3 events per cell, before returning to a new equilibrium not far from that of the control cells, with 6.2/6.4/3.4 events per cell."

Reviewer's comment 4.

See first comment below on discussion. Consider removing the subcellular localization comments from the abstract. They distract from the main points of the paper.

Authors' response 4.

We thank the reviewer for this comment. We have now removed the subcellular localisation comments from the abstract, so as to enhance focus.

Reviewer's comment 5.

Results:

Why is data collected on control cells for only ~250 seconds when it appears that the treated group is imaged for twice as long (combining pre treatment and post treatment)? This opens up the opportunity for confounding variables including photo bleaching and photooxidative stress. Was vehicle added to the control cells at the same time point as the treated cells?

Authors' response 5.

We thank the reviewer for this important point. Indeed, all cells were acquired for a period of 300 seconds. A five-frame moving average was employed in the graphs in Figs 6-10 and is mentioned in the text in line 412.

We also recognise that the difference between the acquisition time between the control and treated cells were not clearly explained. We have now carefully described this in the manuscript (line 345):
"Low laser power was used to ensure that photo bleaching and photooxidative stress was limited during this acquisition period. Since the total acquisition time for the treated cells was 10 minutes (5 minutes before treatment and 5 minutes after treatment), the control cells were also acquired for a total of 10 minutes in order to ensure that photo bleaching was limited (as shown in S4 Fig) and therefore the effects of the treatment on the cell could be interpreted as resulting primarily from the treatment itself. Only the data of the first 5 minutes of the control cells are shown in the results section."

Reviewer's comment 6.

When it comes to statistical analysis, I'm not sure that lumping all of the many time-resolved events together via an average makes the most convincing argument for differences. While it is great and important to show the very high temporal resolution of the method, it would be more interesting and probably more statistically powerful to look at the summation of events over time (on a per cell basis) as opposed to averaging over time. Other alternatives would be analyzing specific time points or the derivative/rate of change.

Authors' response 6.

This is an excellent point, to expand on the statistical analysis beyond the temporal resolution. We have now also added the plots of the cumulative sum of the events over time and included the average as well as the confidence interval between the experimental replicates at each time point in Fig 10B.

Minor critiques:

Reviewer's comment 7.

Figure 6:
The control and post treatment time lapse curves appear identical. Please verify.

Authors' response 7.

We greatly appreciate that the reviewer picked up on this. We apologise for this and have now corrected it in the revised manuscript.

Reviewer's comment 8.

Secondly, the panels at the very bottom employ the use of a line graph that suggests a paired nature to the data. Bar graphs grouped by each event type would make the data easier to interpret to the reader. The error bars in the updated figure should be error between experimental replicates, not error between cells within an experimental replicate. Please add text to clarify that the number of events are per cell or per high powered field.

Authors' response 8.

We have replaced the interval and line graph with grouped bar graphs in Fig 6, as suggested. Furthermore, we have now removed the interval plots that were related to the error between cells within an experimental replicate in Fig 10.

Reviewer's comment 9.

Why are the control events so different from the pre-treated cells? It makes the reader wonder whether there is a high amount of biological variability (which could be addressed with repeat experiments) or a confounding variable has not been addressed in the experimental set up (temperature, CO2, pH?).

Authors' response 9.

We thank the reviewer for this comment. We have now chosen a micrograph that is more representative. Temperature, CO_2 and pH have been tightly controlled (Tokai Hit system), similarly to all acquisition parameters. Low laser power was used in order to ensure photobleaching was limited (as validated in the control cells as shown in S4 Fig).

In addition, we indicate and refer to acquisition systems with fully automated fluidics system, which will be of high value for future high throughput screening and drug discovery, associated with cellular pathology with mitochondrial dysfunction, such as specific Parkin mutations and alike (line 553).

Reviewer's comment 10.

Figure 7-9:
Why is there such a difference in the total number of detected structures in the last frame of untreated versus the first frame of the hydrogen peroxide-treated cells? The appearance of the cell has significantly changed suggesting that time 0 is not exactly the time at which peroxide was administered.

Authors' response 10.

The cell that was selected for Figs 8 and 9 to illustrate the pre-treatment and post-treatment groups unfortunately exhibited the behaviour that the reviewer pointed out. There was in fact some time that elapsed (5-10 seconds) between the end of the pre-treatment time lapse acquisition and the start of the post-treatment time-lapse acquisition. This is a limitation of the experimental setup. The difference in the total number of detected structures was however not consistently observed and we have therefore decided to change the representative cell for the pre-treatment and post-treatment conditions which does not exhibit this behaviour.

Reviewer's comment 11.

Furthermore, the overall cell morphology of the pre-treated and the control cell is completely different. I infer from the 2D representation that the pre-treated cell is more rounded and less adherent to the culture plate which is in contrast to the control which has the absence of mitochondria within the nuclear borders and spindle-shaped morphology and is adjacent to another cell. This suggests a different cell state which in and of itself can cause vastly different responses to treatment as well as baseline cell signaling characteristics. I would verify culture conditions are exactly the same between the groups. However, for representation of data, I would suggest picking cells that appear similar at baseline to show your reader.

Authors' response 11.

We thank the reviewer for this comment. We have now amended the pre- and post-treatment figures entirely and utilized a cell that appears similar in morphology (Figs 8 and 9).

Although our cell line was not synchronised, we now also refer to additional approaches that would minimize cell cycle dependent effects or would allow enhance control over cell geometry (line 528).

Reviewer's comment 12.

Discussion:
I would refrain from making statements about patterns observed that aren't rooted in multiple observations with quantification across biological replicates with statistical testing of your hypothesis. In particular, making claims about sub cellular localization of events in reference to peri nuclear versus periphery when no distance data is presented in the paper may make the reader question your findings. Instead, I would suggest alluding how your method could be used to test those hypotheses in future studies.

Authors' response 12.

We have now edited the manuscript to remove specific reference to the subcellular localisation of events. In line 528 we now allude to the potential to investigate subcellular localisation of events in future studies. We also highlight the possibility to combine MEL with the calculation of other functional mitochondria parameters.

Reviewer's comment 13.

Conclusion:
Other methods (including those you referenced) have achieved deterministic automated qualification of mitochondrial morphology, albeit in in 2D not 3D. I would qualify your closing statements so as to not discount the work of others.

Authors' response 13.

This is an important recommendation; we thank the reviewer for pointing this out. We have now edited this section in lines 534 and 553, so as to not discount previous work, but rather indicate the importance of building collectively upon the existing body of knowledge.

General paper edits.

We have supplied both the revised (clean) manuscript as well as a version indicating all amendments and edits.
